# Risk of chronic kidney disease in young adults with impaired glucose tolerance/impaired fasting glucose: a retrospective cohort study using electronic primary care records

**DOI:** 10.1186/s12882-018-0834-4

**Published:** 2018-02-26

**Authors:** Ferozkhan Jadhakhan, Tom Marshall, Ronan Ryan, Paramjit Gill

**Affiliations:** 1 Primary Care Clinical Sciences, Institute of Applied Health Research, University of Birmingham, Edgbaston, Birmingham, B15 2TT UK; 2 WMS – Social Science and Systems in Health, University of Warwick, Coventry, CV4 7AL UK

**Keywords:** Impaired glucose tolerance, Impaired fasting glucose, Chronic kidney disease, The Health Improvement Network (THIN) database, Type 2 diabetes (T2DM), Read code, Incidence rate ratio (IRR)

## Abstract

**Background:**

The risk of chronic kidney disease (CKD) is known to be elevated in patients with diabetes mellitus but the risk of young adults aged 18 to 40 years with impaired glucose tolerance/impaired fasting glucose (IGT/IFG) developing CKD is not well characterised. Furthermore, progression of IGT/IFG to diabetes and subsequent CKD development is not well understood.

**Methods:**

A retrospective cohort study was undertaken using The Health Improvement Network (THIN) database, a large dataset of electronic patient records. THIN database is jointly managed by IMS Health Real World Evidence Solution (http://www.epic-uk.org/index.html) and In Practice System (InPs). Cases were aged 18 to 40, with a diagnosis of IGT/IFG and registered at a practice contributing to THIN between 2000 and 2015. The study population consisted of 40,092 patients, including 21,454 (53.5%) female and 18,638 (46.5%) male. The median follow-up was approximately 2 years. The outcome was a diagnosis of CKD determined from either clinical coding or laboratory results. For the primary analysis the unadjusted and adjusted relative risk of CKD in IGT/IFG was compared to age, sex and practice matched controls with normoglycaemia. For the secondary analysis we compared the incidence of CKD before to after a diagnosis of type 2 diabetes (T2DM) in the IGT/IFG study cohort.

**Results:**

The Incidence Rate Ratio (IRR) for CKD for IGT/IFG compared to normoglycaemia was 4.0 [95% confidence interval (CI), 3.2 to 5.1, *P* < 0.001]. The adjusted IRR was 2.6 [95% CI, 2.0 to 3.4, *P* < 0.001]. The unadjusted IRR was 8.8 [95% CI, 7.7 to 10.0, *P* < 0.001] after IGT/IFG patients had developed T2DM and the adjusted IRR was 6.3 [95% CI, 5.5 to 7.2, *P* < 0.001].

**Conclusion:**

Our results show that young IGT/IFG subjects are also at higher risk of developing CKD. This risk is modulated by the degree of baseline renal function and glucose tolerance, being higher in those developing T2DM.

**Electronic supplementary material:**

The online version of this article (10.1186/s12882-018-0834-4) contains supplementary material, which is available to authorized users.

## Background

CKD is defined as a gradual loss of kidney function for more than three months with or without kidney damage [1]. It is common, progressive, usually asymptomatic [2] and can co-exist with other conditions [2]. The health burden of CKD is likely to rise because the prevalence of type 2 diabetes (T2DM) has been rapidly increasing worldwide [3]. Although the risk of CKD in patients with diabetes is relatively well established, the risk of young adults aged 18 to 40 years with IGT/IFG developing CKD is not well characterised. IGT/IFG is an intermediate state between normal glucose homeostasis and diabetic hyperglycaemia. Individuals with IGT/IFG have glucose levels higher than normal but not high enough for a diagnosis of diabetes [4]. In a ten year prospective study of 241 individuals with IGT, 15% developed T2DM, 53% reverted to normal glucose tolerance and 22% remained glucose intolerant [5]. Similarly, a 4 year prospective study conducted in 128 South African Indians with IGT at baseline, found that after 4 years, 50.4% progressed to diabetes, 24.8% persisted with IGT and 24.8 reverted to normoglycaemia [6]. There is some evidence that the incidence of CKD is elevated in individuals with IGT/IFG, but this is confined to specific populations [7–9]. Furthermore, the progression of IGT/IFG to T2DM and subsequent CKD development is not well understood. As many studies have used a single determination of glycaemic status at baseline, it is not clear whether the risk of developing a CKD event is confined to people with IGT/IFG who progress to overt diabetes or whether the risk is still increased among people with IGT/IFG even if they never develop diabetes. To address these considerations, we investigated the incidence of CKD in patients with IGT/IFG compared to matched controls with normoglycaemia. This was followed by a comparison of the incidence of CKD before and after a diagnosis of T2DM. The comparison was between the IGT/IFG period and the post T2DM period for all IGT/IFG patients who were in the study cohort.

## Methods

### Study design

This was a population based, retrospective, open cohort study using the electronic health record of patients registered with a family practice contributing to The Health Improvement Network (THIN) database.

### Data source

THIN contains anonymised electronic patient records from over 600 UK general practices. As of September 2014 the database, held 13 million patients, of whom 3.6 million were actively registered with a general practice: more than 85 million person years or approximately 6% of the UK population [10] (http://www.epic-uk.org/our-data/statistics.shtml). General practices contributing data to THIN use Vision patient records computer system which codes clinical data using the 5-byte Read code clinical classification [11]. Drug prescriptions issued can be linked to the British National Formulary [12]. Read code lists used to define the outcomes and conditions of interest can be found in Additional file 1. These lists were based on the Quality and Outcomes Framework (QOF) version (33.0) 2015 rulesets (http://www.hscic.gov.uk/qofbrv33).

### Study cohorts

Individual practices were eligible to contribute patients from the later of one year after installation of Vision and their Acceptable Mortality Rate (AMR date) until the date of the last data collection (maximum February 2015). This ensured that they were using their records system fully and that the observation period for each patient was not overestimated [13]. Individual patients were eligible for inclusion in the study from the latest of the following four dates: practice eligibility date, patient registration date plus one year (to ensure patients did not have the outcome of interest (CKD) prior to entry), age 18 years, and the study start date (January 2000). Patients were followed up until the earliest of the following dates: age 41 years, transfer out of the practice or death, and their practice’s last collection date.

### Cases (IGT/IFG definition)

Cases with an incident diagnosis of IGT/IFG were identified using the earliest of the following events:

1. A Read coded diagnosis of IGT/IFG or Pre-diabetes – (Appendix 1)
2. Reported measurements of:

- HbA1c in the range 42–47 mmol/mol [14, 15]
- Fasting blood glucose (FPG) in the range 6.1–6.9 mmol/L [15, 16]
- Oral Glucose Tolerance Test (OGTT) blood glucose in the range ≥ 7.8 ≤ 11.1 mmol/L [15, 16]

The date of diagnosis of IGT/IFG became the index date.

### Exclusion criteria

1. Patients with an index date prior to their cohort entry date
2. Patients with a diagnosis of diabetes (type 1 or type 2) prior to their index date
3. Patient cohort exit date was within 90 days of their index date
4. Patient diagnosed with diabetes (type 1 or type 2) within 30 days of their index date
5. Patients with a diagnosis of CKD prior to their index date

### Matched controls

Eligible IGT/IFG cases were matched to three normoglycaemic controls (without IGT/IFG or T2DM) on gender, age (within 2 years), and registration at the same family practice at the IGT/IFG case index date. Matched controls patients may not necessarily have a blood test to confirm their glycaemic status, suggesting that their glycaemic status may not be known but are generally assumed to be normoglycaemic. The same inclusion and exclusion criteria were applied to potential matches, except that they were required not to have a diagnosis of IGT/IFG on the index date of the IGT/IFG case.

### Follow-up

IGT/IFG cases and matched patients were followed up until the earliest of the following dates: patient died; left practice; diagnosed with CKD; diagnosed with T2DM; last data collection from practice; or they reached the age of 41. For the secondary analysis IGT/IFG cases were not censored at T2DM diagnosis.

### Outcome (CKD definition)

The outcome of interest was a diagnosis of incident CKD stage 1–5 identified using the earliest of the following events:

1. A clinically coded diagnosis of CKD stage (1–2/3–5) – (Appendix 1) or
2. A single estimated glomerular filtration rate (eGFR) of (≥60 ml/min/1.73m^2^ [CKD stage 1–2]) with additional evidence of kidney disease which include the following:
  A single albumin creatinine ratio (ACR) of (≥3 mg/mmol) [17]
  A single protein creatinine ratio (PCR) of (50 mg/mmol) [17]
  A single urinary albumin/protein loss of 0.5 g/day [17]
3. eGFR< 60 ml/min/1.73 m2 (CKD stage 3–5)

### Covariates

Covariates (risk modifiers) were selected based on their biological plausibility and previously published epidemiological evidence [18, 19]. These covariates were age, hypertension, heart failure (HF), atrial fibrillation (AF), cardiovascular disease (CVD) and exposure to a non-steroidal anti-inflammatory drug (NSAID). The latter was defined as a prescription during the year before the index date. All the other covariates were recorded at any time before the index date.

### Ethics approval

THIN database (data collection process) obtained ethical approval from the South East Multi-centre Research Ethics Committee (reference number: 07/H1102/103). The protocol for this study was reviewed and approved in June 2014 (study reference: 14–038) by the THIN Scientific Review Committee (SCR).

### Statistical analysis

- Descriptive statistics

All statistical analyses were conducted using Stata version 13.1 (Stata Corp, College Station Texas, USA). Appropriate descriptive statistics were used to summarise the covariates and matching characteristics for those exposed and unexposed to IGT/IFG. Categorical variables were investigated using (Chi-square [*x*^2^]) test and continuous variables were analysed using t-test. Since, length of follow-up had a highly skewed distribution Mann-Whitney U test was used to compare groups. For categorical variable (e.g., ethnicity) and continuous variables (e.g., BMI) a separate ‘missing’ category was created for those with missing data.

- Calculating incidence rate of CKD:
  1. IGT/IFG compared to normoglycaemia

Adjusted and unadjusted IRRs for CKD in patients aged 18 to 40 years for the period January 2000 to February 2015 was estimated. Poisson regression was used to adjust for patient level covariates (age, hypertension, heart failure, atrial fibrillation, cardiovascular disease and steroid treatment).

2. IGT/IFG period compared to post T2DM period

Adjusted and unadjusted IRRs for CKD in patients aged 18 to 40 years for the period January 2000 to February 2015 was estimated. Poisson regression was used to adjust for patient level covariates.

## Results

### Characteristics of matched cohort study population

The study population consisted of 10,561 patients with IGT/IFG cases and 29,531 matched controls (Table 1). For both cases and controls median age at index date was 35 years, 54% were female and median follow-up was approximately 1.7 years. Compared to patients with normoglycaemia, patients with IGT/IFG were more likely to be of South Asian ethnicity, from areas of greater deprivation, and to have a BMI of 30.0 kg/m^2^ or higher. Atrial fibrillation, heart failure and cardiovascular disease, hypertension and prescriptions for non-steroidal anti-inflammatory drugs were more frequent in IGT/IFG than normoglycaemic patients. During follow-up 11.9% of IGT/IFG and 0.4% of the normoglycaemia cohorts developed T2DM. In addition 1.7% of IGT/IFG cases were diagnosed with CKD compared to 0.4% in the normoglycaemia cohort.

**Table 1:** Characteristics of the matched cohort (IGT/IFG compared to normoglycaemia)

	IGT/IFG [*N* = 10,561 (26%)]		Normoglycaemia [*N* = 29,531 (74%)]		*P-*Value
Age, mean [SD]	33.9	[5.3]	33.8	[5.4]	0.015^††^
Age, median [IQR]	35	[31, 38]	35	[31, 38]	
Female, No. (%)	5659	(53.6)	15,795	(53.5)	0.863^†^
Years of follow-up, mean [SD]	2.3	[2.1]	2.3	[2.0]	0.684^†††^
Years of follow-up, median [IQR]	1.68	[0.81, 3.16]	1.67	[0.79, 3.14]	
Ethnicity, No. (%)					
White	3644	(34.5)	11,305	(38.3)	< 0.001^†^
Black	392	(3.7)	784	(2.7)	
Asian	1483	(14.0)	1902	(6.4)	
Chinese	32	(0.3)	102	(0.4)	
Mixed	63	(0.6)	219	(0.7)	
Others	130	(1.2)	333	(1.1)	
Unknown	4817	(45.6)	14,886	(50.5)	
Deprivation quintile, No. (%)					
(least deprived) 1	1509	(14.3)	5229	(17.7)	< 0.001^†^
2	1575	(14.9)	4985	(16.9)	
3	2177	(20.6)	6149	(20.8)	
4	2463	(23.3)	6319	(21.4)	
(most deprived) 5	2232	(21.1)	5020	(17.0)	
Unknown	605	(5.7)	1829	(6.2)	
CVD at index date, No. (%)	190	(1.8)	274	(0.9)	< 0.001^†^
HF at index date, No. (%)	29	(0.30)	21	(0.07)	< 0.001^†^
AF at index date, No. (%)	17	(0.16)	21	(0.07)	0.010^†^
Hypertension at index date, No. (%)	1112	(10.5)	548	(1.9)	< 0.001^†^
Prescription of NSAID, No. (%)	200	(1.9)	120	(0.4)	< 0.001^†^
BMI (kg/m^2^), No. (%)					
< 20	332	(3.1)	2191	(7.4)	< 0.001^††^
20–24.9	1542	(14.6)	9452	(32.0)	
25–29.9	2420	(22.9)	7028	(23.8)	
30–34.9	2104	(19.9)	2868	(9.7)	
35–39.9	1585	(15.0)	1071	(3.6)	
≥ 40	1575	(15.0)	644	(2.2)	
Unknown	1003	(9.5)	6277	(21.2)	
BMI (kg/m^2^), mean [SD]	26.0	[5.7]	32.1	[8.2]	< 0.001^††^
BMI (kg/m^2^), median [IQR]	31	[26, 37.1]	24.9	[22.1, 28.7]	
T2DM during follow-up, No. (%)	1262	(11.9)	124	(0.4)	< 0.001^†^
CKD during follow-up, No. (%)	182	(1.7)	126	(0.4)	< 0.001^†^
Country, No. (%)					
England	8516	(80.6)	23,661	(80.0)	0.688^†^
Northern Ireland	270	(2.6)	791	(2.7)	
Scotland	804	(7.6)	2321	(7.9)	
Wales	971	(9.2)	2758	(9.3)	

Values are numbers (percentages) unless otherwise specified. *P*-values are from *X*^2^ test (^†^) for proportions or t-test (^††^) for continuous variables (age, BMI) and Mann-Whitney U test (^†††^) for follow-up

*Abbreviations*: *CVD* cardiovascular disease, *HF* heart failure, *AF* atrial fibrillation, *NSAID* non-steroidal anti-inflammatory drug, *BMI* body mass index, *IQR* interquartile range

Table 2 provides information about the number of CKD cases first recoded via Read code and first recorded via biomedical data in the IGT/IFG cohort.

**Table 2:** Cases of CKD first recorded by Read code and first recorded by biomedical data in the IGT/IFG cohort

CKD results	Read code	Biomedical data	Total
CKD cases recorded	26	156	182

*Abbreviation*: *CKD* chronic kidney disease

### Incidence rate of CKD in persons aged 18 to 40 years with IGT/IFG compared to those with normoglycaemia

The unadjusted incidence of CKD per 100,000 person-years in persons with IGT/IFG was 4 times higher than individuals with normoglycaemia [IRR: 4.0; 95% CI, 3.2 to 5.1, *P* < 0.001]. After adjustment for age, sex, ethnic group, deprivation quintile, BMI categories, cardiovascular disease, heart failure, atrial fibrillation, hypertension and steroid use, incidence was reduced to approximately 3 times higher [IRR: 2.6; 95% CI, 2.0 to 3.4, *P* < 0.001] in persons with IGT/IFG (Table 3). In the adjusted analysis there was an increased incidence of CKD for each additional year of age at IGT/IFG diagnosis [IRR: 1.1; 95% CI, 1.0 to 1.1, *P* < 0.001] and an increased incidence in patients with a diagnosis of hypertension [IRR: 3.1; 95% CI, 2.3 to 4.2, *P* < 0.001].

**Table 3:** Adjusted incidence rate of CKD in IGT/IFG compared to normoglycaemia

Predictors		IRR	95% (CI)	*P-*Value
IGT/IFG vs normoglycaemia		2.6	(2.0, 3.4)	< 0.001
Female		1.1	(0.9, 1.4)	0.425
Age (in years)		1.1	(1.0, 1.1)	< 0.001
Ethnicity				
White	Reference	1.0		
Asian		1.2	(0.8, 1.7)	0.389
Black		1.0	(0.6, 1.9)	0.955
Chinese		1.2	(0.2, 8.7)	0.845
Mixed		0.5	(0.1, 3.3)	0.439
Other		1.0	(0.4, 2.8)	0.970
Missing		0.7	(0.5, 0.8)	0.001
BMI (kgs/m^2^)				
< 20	Reference	1.0		
20–24.9		1.9	(0.9, 4.5)	0.127
25–29.9		2.5	(1.1, 5.8)	0.029
30–34.9		3.2	(1.4, 7.5)	0.006
35–39.9		2.6	(1.1, 6.2)	0.037
≥ 40		3.2	(1.3, 7.6)	0.010
Missing		1.4	(0.6, 3.5)	0.452
Deprivation quintile				
Least deprived 1	Reference	1.0		
2		0.8	(0.5, 1.3)	0.394
3		1.1	(0.8, 1.6)	0.571
4		0.9	(0.6, 1.4)	0.756
Most deprived 5		1.3	(0.9, 1.9)	0.123
Missing		1.4	(0.8, 2.3)	0.256
CVD		0.9	(0.4, 2.3)	0.905
HF		0.8	(0.1, 6.2)	0.872
AF		2.9	(0.4, 21.0)	0.285
Hypertension		3.1	(2.3, 4.2)	< 0.001
Steroid		1.1	(0.9, 1.5)	0.404

*Abbreviations*: *BMI* body mass index, *CVD* cardiovascular disease, *HF* heart failure, *AF* atrial fibrillation, *CI* confidence interval

### Incidence rate of CKD in persons aged 18 to 40 years before and after T2DM

The unadjusted incidence of CKD in patients with IGT/IFG after T2DM diagnosis was approximately 9 times higher [IRR: 8.8; 95% CI, 7.7 to 10.0, *P* < 0.001] compared to patients with IGT/IFG. After adjustment with potential confounders, the incidence of CKD remained high [IRR: 6.3; 95% CI, 5.5 to 7.2, *P* < 0.001] in patients who progressed to T2DM compared to those with IGT/IFG (Table 4). Of the adjusted covariates there was an increased incidence of CKD for each additional year of age at T2DM diagnosis [IRR: 1.3 (95% CI, 1.2 to 1.3, *P* < 0.001] the incidence of CKD was also increased in patients with a diagnosis of hypertension [IRR: 1.6; 95% CI, 1.4 to 1.9, *P* < 0.001].

**Table 4:** Adjusted incidence rate of CKD in T2DM compared to IGT/IFG

Predictors		IRR	95% (CI)	*P-*Value
T2DM vs IGT/IFG		6.3	(5.5, 7.2)	< 0.001
Female		1.2	(1.0, 1.4)	0.008
Age (in years)		1.3	(1.2, 1.3)	< 0.001
Ethnicity				
White	Reference	1.0		
Asian		0.8	(0.6, 1.0)	0.096
Black		0.8	(0.5, 1.2)	0.279
Chinese		0.6	(0.1, 4.2)	0.601
Mixed		0.9	(0.4, 2.2)	0.856
Other		0.8	(0.5, 1.5)	0.538
Missing		0.8	(0.7, 0.9)	0.001
BMI (kgs/m^2^)				
< 20	Reference	1.0		
20–24.9		2.1	(0.8, 5.2)	0.110
25–29.9		2.6	(1.1, 6.4)	0.035
30–34.9		3.2	(1.3, 7.8)	0.010
35–39.9		3.3	(1.3, 8.0)	0.009
≥ 40		2.8	(1.1, 6.7)	0.025
Missing		1.9	(0.7, 4.8)	0.181
Deprivation quintile				
Least deprived 1	Reference	1.0		
2		0.9	(0.7, 1.1)	0.390
3		1.0	(0.9, 1.2)	0.947
4		0.9	(0.7, 1.1)	0.349
Most deprived 5		0.7	(0.6, 0.9)	0.008
Missing		1.0	(0.7, 1.4)	0.830
CVD		1.3	(0.9, 2.0)	0.231
HF		0.7	(0.2, 2.2)	0.514
AF		2.0	(0.7, 5.4)	0.174
Hypertension		1.6	(1.4, 1.9)	< 0.001
Steroid		1.0	(0.8, 1.1)	0.675

*Abbreviations*: *BMI* body mass index, *CVD* cardiovascular disease, *HF* heart failure, *AF* atrial fibrillation, *CI* confidence interval

## Discussion

The objective of this study was to investigate the incidence of CKD in a retrospective matched cohort analysis using the THIN database and found that young adults with IGT/IFG were four times more at risk of developing CKD than individuals with normoglycaemia. After adjusting for age, sex, ethnic group, deprivation quintile, BMI categories, cardiovascular disease, heart failure, atrial fibrillation, hypertension and NSAID, the effect of CKD risk was attenuated but was still 2.6 times higher in individuals with IGT/IFG than those with normoglycaemia. Among the modifiable risk factors, hypertension was consistently linked to higher incidence of CKD. Individuals progressing from IGT/IFG to T2DM were approximately nine times more likely to be diagnosed with CKD following diabetes diagnosis than individuals with IGT/IFG. After adjustment for potential confounders, incidence of CKD was attenuated but still showed a significant association. The incidence was reduced to approximately 7 times higher in T2DM than the IGT/IFG cohort.

### Strengths and limitations

#### Strengths

The strengths include the large sample size of the database (THIN contains medical records of over 12 million patients) which is approximately 6% of the UK population [10]. Generalisability was ensured by the fact that the dataset includes similar patients to those seen in primary care across the UK. This reflects the fact that data are collected in a non-interventional fashion during routine clinical practice. Internal validity (reducing the effect of confounders) was ensured by matching patient’s age, sex and general practice. Although the study was retrospective, data were collected prospectively, hence eliminating recall bias. Ascertainment of exposure and outcome of interest was carried out by both Read coded diagnosis and clinical measurements (e.g. HbA1c, ACR lab results). Additionally, the completeness of lab results has significantly improved since it is now directly fed into GP electronic patient records instead of entered manually. Therefore, improving ascertainment of IGT/IFG and CKD for this study and providing a reliable estimate of incident CKD in young adults with IGT/IFG. This occurred during the study period (2000–2015); earlier years may have had under reporting.

#### Limitations

The main limitation relates to the potential misclassification of patients with IGT/IFG (i.e. patients are identified as normoglycaemic when in fact they had IGT/IFG but were not identified because they had no blood tests). This would dilute the relationship between IGT/IFG and CKD because some of the controls have IGT/IFG and the likely association between IGT/IFG and risk of CKD may be reduced. Additionally, there is also potential for misclassification of patients with CKD (i.e. patients Read coded as CKD but with no laboratory reported evidence of reduced eGFR and patients with laboratory evidence of CKD but with no Read code labelling of CKD). However, for this study both laboratory evidence of reduced eGFR and Read code were used to identify incident CKD. There is incomplete data on some important confounding variables (e.g., ethnicity, BMI). Additionally, before automated reporting and recording, lab results were entered manually and in some cases only abnormal results may have been entered into the GP system: this could underestimate the incidence of CKD. There is also potential for ascertainment bias which may arise when patients who have frequent laboratory tests may be more likely to have both IGT/IFG and CKD diagnosed. Furthermore, the incidence and prevalence of recorded CKD depends on general practitioners (GPs) doing blood tests. This may underestimate the frequency of CKD and may also be biased because GPs are more likely to do blood tests for CKD in patients in whom they suspect CKD. This includes those with diabetes, hypertension, certain ethnic groups and those with IGT/IFG. This is reflected in the number of CKD recorded via Read code and laboratory measurements.

### Comparison with other studies

The results of this study appear to be consistent with previous studies showing an elevated risk of CKD in adults with IGT/IFG [8, 20, 21]. Watanabe and colleagues [9] in the Niigata Preventive Medicine Study, examining the risk of CKD in individual components of metabolic syndrome, found the incidence of IGT/IFG was approximately twice as high in subjects with IGT/IFG as those with normoglycaemia. Furthermore, a retrospective study, using data from the Framingham Offspring cohort study examined the risk of CKD development in non-diabetic patients, found that patients with IGT/IFG were approximately two times more likely to develop CKD and those with diabetes were approximately four times more at risk of CKD [7]. Additionally, A prospective study, using data from the Italian Association of Clinical Diabetologists (Associazione Medici Diabetologi, AMD) initiative, examined risk factors associated with the development of CKD in patients aged (≥18 years) with type 2 diabetes and without CKD or albuminuria at baseline. Patients were followed up between January 1, 2004 and June 30, 2008. During follow-up, patients who showed reduced kidney function and albuminuria were older males with poor glycaemic control, higher systolic blood pressure, hyperlipidaemia and on hypertensive and lipid lowering drugs [22]. The risk factors identified in this study are similar to those identified in the current study. The Framingham study contained data of 2398 patients aged (≥28 years) and 47% were male compared to a study population of over 40,000 individuals aged 18 to 40 years in the current study. The gender distributions were similar in both studies. The follow-up period in the Yamagata and colleagues study was 10 years compared a shorter length of follow-up for individual patients in the current study (median 2 years approximately). In their study, Yamagata and colleagues included 113, 764 individuals aged (≥40 years) and 33% were male compared to a study population of over 40,000 individuals aged 18 to 40 years and 46.4% male in the current study. These studies, however, did not report separate results in persons aged 18 to 40 years with IGT/IFG and risk of CKD, but rather results were presented for the population as a whole.

## Conclusion

To the best of our knowledge, this study was the first population based study to address this question utilising data from a large generalisable cohort of general practices and their patients from across the UK. It used contemporary data, to investigate the incidence of CKD in young adults aged 18 to 40 years with IGT/IFG compared to those with normoglycaemia. This study provides evidence of an increased risk of CKD amongst young adults with IGT/IFG. Analyses revealed that patients progressing from IGT/IFG to T2DM are at an even higher risk of developing CKD, providing further opportunity for individuals with IGT/IFG to consider lifestyle modification and have regular follow-up check of their kidney function to detect CKD at an early stage. Additionally, among the modifiable risk factors, hypertension was consistently linked to higher incidence of CKD. The result of this study shows the sizeable adverse effect of IGT/IFG on the development of CKD, and if left undetected may cause serious long term health issues and should be addressed at an early stage to reduce the future burden of CKD in patients with IGT/IFG.

## Additional file

Additional file 1: **Appendix.** Read code lists. (DOCX 89 kb)

## Abbreviations

ACR: Albumin creatinine ratio
AF: Atrial fibrillation
AMR: Acceptable mortality recording
BMI: Body Mass Index
BNF: British National Formulary
CHD: Coronary heart disease
CI: Confidence interval
CKD: Chronic kidney disease
CVD: Cardiovascular disease
EGFR: Estimated glomerular filtration rate
FPG: Fasting plasma glucose
HbA1c: Glycosylated haemoglobin
HF: Heart failure
HR: Hazards ratio
IFG: Impaired fasting glucose
IGR: Impaired glucose regulation
IGT: Impaired glucose tolerance
InPs: In Practice Systems
IQR: Interquartile range
IR: Incidence rate
IRR: Incidence Rate Ratio
NIHR: National Institute for Health and Care Excellence
NR: Not reported
NSAIDS: Non-steroidal anti-inflammatory drugs
OGTT: Oral Glucose Tolerance Test
PCR: Protein creatinine ratio
PYR: Person years
QOF: Quality and Outcomes Framework
RR: Relative risk
SCR: Serum Creatinine
SD: Standard deviation
T2DM: Type 2 Diabetes
THIN: The health improvement network database
UK: United Kingdom
