# Supplementary material for: Risk of chronic kidney disease in young adults with impaired glucose tolerance/impaired fasting glucose: a retrospective cohort study using electronic primary care records
Source: BMC Nephrol. 2018 Feb 26;19:42. doi: 10.1186/s12882-018-0834-4 (PMC6389049; doi:10.1186/s12882-018-0834-4)
Supplement: Supplementary file 1 — Appendix. Read code lists. (DOCX 89 kb) [file 12882_2018_834_MOESM1_ESM.docx]

**Additional file 1: Appendix**

**Read codes used to identify cases of IGT/IFG**

| **Read code** | **Description** |
| --- | --- |
| 44V2.00 | Glucose tolerance test impaired |
| C11y200 | Impaired glucose tolerance |
| C11y300 | Impaired fasting glycaemia |
| C11y400 | Impaired glucose regulation |
| C11y500 | Pre-diabetes |
| 6AC..00 | Review of impaired glucose tolerance |
| 9NS0400 | Referral for impaired glucose tolerance management offered |
| R102.00 | [D]Glucose tolerance test abnormal |
| R102.11 | [D]Prediabetes |
| R102.12 | [D]Impaired glucose tolerance test |
| R10D000 | [D]Impaired fasting glycaemia |
| R10D011 | [D]Impaired fasting glucose |
| R10E.00 | [D]Impaired glucose tolerance |
| C313500 | Glucose intolerance |

Abbreviation: [D], diagnosis

**Read code used to identify cases of T2DM**

**Note:** This list include codes which do not specify diabetes type

| **Read Code** | **Description** |
| --- | --- |
| C10..00 | Diabetes mellitus |
| C109J00 | Insulin treated Type 2 diabetes mellitus |
| C109K00 | Hyperosmolar non-ketotic state in type 2 diabetes mellitus |
| C10C.00 | Diabetes mellitus autosomal dominant |
| C10D.00 | Diabetes mellitus autosomal dominant type 2 |
| C10F.00 | Type 2 diabetes mellitus |
| C10F.11 | Type II diabetes mellitus |
| C10F000 | Type 2 diabetes mellitus with renal complications |
| C10F011 | Type II diabetes mellitus with renal complications |
| C10F100 | Type 2 diabetes mellitus with ophthalmic complications |
| C10F111 | Type II diabetes mellitus with ophthalmic complications |
| C10F200 | Type 2 diabetes mellitus with neurological complications |
| C10F211 | Type II diabetes mellitus with neurological complications |
| C10F300 | Type 2 diabetes mellitus with multiple complications |
| C10F311 | Type II diabetes mellitus with multiple complications |
| C10F400 | Type 2 diabetes mellitus with ulcer |
| C10F411 | Type II diabetes mellitus with ulcer |
| C10F500 | Type 2 diabetes mellitus with gangrene |
| C10F511 | Type II diabetes mellitus with gangrene |
| C10F600 | Type 2 diabetes mellitus with retinopathy |
| C10F611 | Type II diabetes mellitus with retinopathy |
| C10F700 | Type 2 diabetes mellitus - poor control |
| C10F711 | Type II diabetes mellitus - poor control |
| C10F900 | Type 2 diabetes mellitus without complication |
| C10F911 | Type II diabetes mellitus without complication |
| C10FA00 | Type 2 diabetes mellitus with mononeuropathy |
| C10FA11 | Type II diabetes mellitus with mononeuropathy |
| C10FB00 | Type 2 diabetes mellitus with polyneuropathy |
| C10FB11 | Type II diabetes mellitus with polyneuropathy |
| C10FC00 | Type 2 diabetes mellitus with nephropathy |
| C10FC11 | Type II diabetes mellitus with nephropathy |
| C10FD00 | Type 2 diabetes mellitus with hypoglycaemic coma |
| C10FD11 | Type II diabetes mellitus with hypoglycaemic coma |
| C10FE00 | Type 2 diabetes mellitus with diabetic cataract |
| C10FE11 | Type II diabetes mellitus with diabetic cataract |
| C10FF00 | Type 2 diabetes mellitus with peripheral angiopathy |
| C10FF11 | Type II diabetes mellitus with peripheral angiopathy |
| C10FG00 | Type 2 diabetes mellitus with arthropathy |
| C10FG11 | Type II diabetes mellitus with arthropathy |
| C10FH00 | Type 2 diabetes mellitus with neuropathic arthropathy |
| C10FH11 | Type II diabetes mellitus with neuropathic arthropathy |
| C10FJ00 | Insulin treated Type 2 diabetes mellitus |
| C10FJ11 | Insulin treated Type II diabetes mellitus |
| C10FK00 | Hyperosmolar non-ketotic state in type 2 diabetes mellitus |
| C10FK11 | Hyperosmolar non-ketotic state in type II diabetes mellitus |
| C10FL00 | Type 2 diabetes mellitus with persistent proteinuria |
| C10FL11 | Type II diabetes mellitus with persistent proteinuria |
| C10FM00 | Type 2 diabetes mellitus with persistent microalbuminuria |
| C10FM11 | Type II diabetes mellitus with persistent microalbuminuria |
| C10FN00 | Type 2 diabetes mellitus with ketoacidosis |
| C10FN11 | Type II diabetes mellitus with ketoacidosis |
| C10FP00 | Type 2 diabetes mellitus with ketoacidotic coma |
| C10FP11 | Type II diabetes mellitus with ketoacidotic coma |
| C10FQ00 | Type 2 diabetes mellitus with exudative maculopathy |
| C10FQ11 | Type II diabetes mellitus with exudative maculopathy |
| C10FR00 | Type 2 diabetes mellitus with gastroparesis |
| C10FR11 | Type II diabetes mellitus with gastroparesis |
| C10FS00 | Maternally inherited diabetes mellitus |
| C10G.00 | Secondary pancreatic diabetes mellitus |
| C10G000 | Secondary pancreatic diabetes mellitus without complication |
| C10H.00 | Diabetes mellitus induced by non-steroid drugs |
| C10H000 | Diabetes Mellitus induced by non-steroid drugs without complication |
| C10M.00 | Lipoatrophic diabetes mellitus |
| C10M000 | Lipoatrophic diabetes mellitus without complication |
| C10N.00 | Secondary diabetes mellitus |
| C10N000 | Secondary diabetes mellitus without complication |
| C10N100 | Cystic fibrosis related diabetes mellitus |
| C10P.00 | Diabetes mellitus in remission |
| C10P100 | Type II diabetes mellitus in remission |
| C10P111 | Type 2 diabetes mellitus in remission |
| PKyP.00 | Diabetes insipidus,diabetes mellitus,optic atrophy and deafness |

**Read code used to identify cases of CKD**

| **Read code** | **Description** |
| --- | --- |
| 1Z12.00 | Chronic kidney disease stage 3 |
| 1Z13.00 | Chronic kidney disease stage 4 |
| 1Z14.00 | Chronic kidney disease stage 5 |
| 1Z15.00 | Chronic kidney disease stage 3A |
| 1Z16.00 | Chronic kidney disease stage 3B |
| 1Z1B.00 | Chronic kidney disease stage 3 with proteinuria |
| 1Z1B.11 | CKD stage 3 with proteinuria |
| 1Z1C.00 | Chronic kidney disease stage 3 without proteinuria |
| 1Z1C.11 | CKD stage 3 without proteinuria |
| 1Z1D.00 | Chronic kidney disease stage 3A with proteinuria |
| 1Z1D.11 | CKD stage 3A with proteinuria |
| 1Z1E.00 | Chronic kidney disease stage 3A without proteinuria |
| 1Z1E.11 | CKD stage 3A without proteinuria |
| 1Z1F.00 | Chronic kidney disease stage 3B with proteinuria |
| 1Z1F.11 | CKD stage 3B with proteinuria |
| 1Z1G.00 | Chronic kidney disease stage 3B without proteinuria |
| 1Z1G.11 | CKD stage 3B without proteinuria |
| 1Z1H.00 | Chronic kidney disease stage 4 with proteinuria |
| 1Z1H.11 | CKD stage 4 with proteinuria |
| 1Z1J.00 | Chronic kidney disease stage 4 without proteinuria |
| 1Z1J.11 | CKD stage 4 without proteinuria |
| 1Z1K.00 | Chronic kidney disease stage 5 with proteinuria |
| 1Z1K.11 | CKD stage 5 with proteinuria |
| 1Z1L.00 | Chronic kidney disease stage 5 without proteinuria |
| 1Z1L.11 | CKD stage 5 without proteinuria |
| 1Z1N. | CKD with GFR category G1 & albuminuria category A2 |
| 1Z1P. | CKD with GFR category G1 & albuminuria category A3 |
| 1Z1R. | CKD with GFR category G2 & albuminuria category A2 |
| 1Z1S. | CKD with GFR category G2 & albuminuria category A3 |
| 1Z1T. | CKD with GFR category G3a & albuminuria category A1 |
| 1Z1V. | CKD with GFR category G3a & albuminuria category A2 |
| 1Z1W. | CKD with GFR category G3a & albuminuria category A3 |
| 1Z1X. | CKD with GFR category G3b & albuminuria category A1 |
| 1Z1Y. | CKD with GFR category G3b & albuminuria category A2 |
| 1Z1Z. | CKD with GFR category G3b & albuminuria category A3 |
| 1Z1a. | CKD with GFR category G4 & albuminuria category A1 |
| 1Z1b. | CKD with GFR category G4 & albuminuria category A2 |
| 1Z1c. | CKD with GFR category G4 & albuminuria category A3 |
| 1Z1d. | CKD with GFR category G5 & albuminuria category A1 |
| 1Z1e. | CKD with GFR category G5 & albuminuria category A2 |
| 1Z1f. | CKD with GFR category G5 & albuminuria category A3 |
| K053 | CKD (Stage 3) |
| K054 | CKD (Stage 4) |
| K055 | CKD (Stage 5) |
| 1Z10.00 | Chronic kidney disease stage 1 |
| 1Z11.00 | Chronic kidney disease stage 2 |
| 1Z17.11 | CKD stage 1 with proteinuria |
| 1Z18.00 | Chronic kidney disease stage 1 without proteinuria |
| 1Z18.11 | CKD stage 1 without proteinuria |
| 1Z19.00 | Chronic kidney disease stage 2 with proteinuria |
| 1Z19.11 | CKD stage 2 with proteinuria |
| 1Z1A.00 | Chronic kidney disease stage 2 without proteinuria |
| 1Z1A.11 | CKD stage 2 without proteinuria |
| K051.00 | Chronic kidney disease stage 1 |
| K052.00 | Chronic kidney disease stage 2 |
| 1Z1M. | CKD with GFR category G1 & albuminuria category A1 |
| 46TC.00 | Urine albumin: creatinine ratio |
| 44lD.00 | Urine protein: creatinine ratio |
| 44J7.00 | Albumin: creatinine ratio |

**Read codes used to identify cases of atrial fibrillation**

| **Read code** | **Description** |
| --- | --- |
| G573.00 | Atrial fibrillation and flutter |
| G573000 | Atrial fibrillation |
| G573200 | Paroxysmal atrial fibrillation |
| G573300 | Non-rheumatic atrial fibrillation |
| G573400 | Permanent atrial fibrillation |
| G573500 | Persistent atrial fibrillation |
| G573z00 | Atrial fibrillation and flutter NOS |

Abbreviation: NOS, not otherwise specified; GFR, glomerular filtration rate

**Read codes used to identify cases with hypertension**

| **Read code** | **Description** |
| --- | --- |
| G2...00 | Hypertensive disease |
| G20..00 | Essential hypertension |
| G20..11 | High blood pressure |
| G20..12 | Primary hypertension |
| G200.00 | Malignant essential hypertension |
| G201.00 | Benign essential hypertension |
| G202.00 | Systolic hypertension |
| G203.00 | Diastolic hypertension |
| G20z.00 | Essential hypertension NOS |
| G20z.11 | Hypertension NOS |
| G21..00 | Hypertensive heart disease |
| G210.00 | Malignant hypertensive heart disease |
| G210000 | Malignant hypertensive heart disease without CCF |
| G210100 | Malignant hypertensive heart disease with CCF |
| G210z00 | Malignant hypertensive heart disease NOS |
| G211.00 | Benign hypertensive heart disease |
| G211000 | Benign hypertensive heart disease without CCF |
| G211100 | Benign hypertensive heart disease with CCF |
| G211z00 | Benign hypertensive heart disease NOS |
| G21z.00 | Hypertensive heart disease NOS |
| G21z000 | Hypertensive heart disease NOS without CCF |
| G21z011 | Cardiomegaly - hypertensive |
| G21z100 | Hypertensive heart disease NOS with CCF |
| G21zz00 | Hypertensive heart disease NOS |
| G22..00 | Hypertensive renal disease |
| G22..11 | Nephrosclerosis |
| G220.00 | Malignant hypertensive renal disease |
| G221.00 | Benign hypertensive renal disease |
| G222.00 | Hypertensive renal disease with renal failure |
| G22z.00 | Hypertensive renal disease NOS |
| G2 2z.11 | Renal hypertension |
| G23..00 | Hypertensive heart and renal disease |
| G230.00 | Malignant hypertensive heart and renal disease |
| G231.00 | Benign hypertensive heart and renal disease |
| G232.00 | Hypertensive heart&renal dis wth (congestive) heart failure |
| G233.00 | Hypertensive heart and renal disease with renal failure |
| G234.00 | Hyperten heart&renal dis+both(congestv)heart and renal fail |
| G23z.00 | Hypertensive heart and renal disease NOS |
| G24..00 | Secondary hypertension |
| G240.00 | Secondary malignant hypertension |
| G240z00 | Secondary malignant hypertension NOS |
| G241.00 | Secondary benign hypertension |
| G241z00 | Secondary benign hypertension NOS |
| G244.00 | Hypertension secondary to endocrine disorders |
| G24z.00 | Secondary hypertension NOS |
| G24z000 | Secondary renovascular hypertension NOS |
| G24zz00 | Secondary hypertension NOS |
| G25..00 | Stage 1 hypertension (NICE - Nat Ins for Hth Clin Excl 2011) |
| G25..11 | Stage 1 hypertension |
| G250.00 | Stage 1 hyperten (NICE 2011) without evidence end organ damage |
| G251.00 | Stage 1 hyperten (NICE 2011) with evidence end organ damage |
| G26..00 | Severe hypertension (Nat Inst for Health Clinical Excl 2011) |
| G26..11 | Severe hypertension |
| G28..00 | Stage 2 hypertension (NICE - Nat Ins for Hth Clin Excl 2011) |
| G2y..00 | Other specified hypertensive disease |
| G2z..00 | Hypertensive disease NOS |
| Gyu2. | [X]Hypertensive diseases |
| Gyu20. | [X]Other secondary hypertension |

Abbreviations: NOS, not otherwise specified; CCF, congestive cardiac failure

**Read codes used to identify cases of cardiovascular disease**

**Note:** This list includes CHD, PAD, stroke and TIA combined to create a single CVD group.

| **Read code** | **Description** |
| --- | --- |
| CHD codes |  |
| G3...00 | Ischaemic heart disease |
| G3...11 | Arteriosclerotic heart disease |
| G3...12 | Atherosclerotic heart disease |
| G3...13 | IHD - Ischaemic heart disease |
| G30..00 | Acute myocardial infarction |
| G30..11 | Attack - heart |
| G30..12 | Coronary thrombosis |
| G30..13 | Cardiac rupture following myocardial infarction (MI) |
| G30..14 | Heart attack |
| G30..15 | MI - acute myocardial infarction |
| G30..16 | Thrombosis - coronary |
| G30..17 | Silent myocardial infarction |
| G300.00 | Acute anterolateral infarction |
| G301.00 | Other specified anterior myocardial infarction |
| G301000 | Acute anteroapical infarction |
| G301100 | Acute anteroseptal infarction |
| G301z00 | Anterior myocardial infarction NOS |
| G302.00 | Acute inferolateral infarction |
| G303.00 | Acute inferoposterior infarction |
| G304.00 | Posterior myocardial infarction NOS |
| G305.00 | Lateral myocardial infarction NOS |
| G306.00 | True posterior myocardial infarction |
| G307.00 | Acute subendocardial infarction |
| G307000 | Acute non-Q wave infarction |
| G307100 | Acute non-ST segment elevation myocardial infarction |
| G308.00 | Inferior myocardial infarction NOS |
| G309.00 | Acute Q-wave infarct |
| G30B.00 | Acute posterolateral myocardial infarction |
| G30X.00 | Acute transmural myocardial infarction of unspecif site |
| G30X000 | Acute ST segment elevation myocardial infarction |
| G30y.00 | Other acute myocardial infarction |
| G30y000 | Acute atrial infarction |
| G30y100 | Acute papillary muscle infarction |
| G30y200 | Acute septal infarction |
| G30yz00 | Other acute myocardial infarction NOS |
| G30z.00 | Acute myocardial infarction NOS |
| G31..00 | Other acute and subacute ischaemic heart disease |
| G310.11 | Dressler's syndrome |
| G311.00 | Preinfarction syndrome |
| G311.11 | Crescendo angina |
| G311.12 | Impending infarction |
| G311.13 | Unstable angina |
| G311.14 | Angina at rest |
| G311000 | Myocardial infarction aborted |
| G311011 | MI - myocardial infarction aborted |
| G311100 | Unstable angina |
| G311200 | Angina at rest |
| G311300 | Refractory angina |
| G311400 | Worsening angina |
| G311500 | Acute coronary syndrome |
| G311z00 | Preinfarction syndrome NOS |
| G312.00 | Coronary thrombosis not resulting in myocardial infarction |
| G31y.00 | Other acute and subacute ischaemic heart disease |
| G31y000 | Acute coronary insufficiency |
| G31y100 | Microinfarction of heart |
| G31y200 | Subendocardial ischaemia |
| G31y300 | Transient myocardial ischaemia |
| G31yz00 | Other acute and subacute ischaemic heart disease NOS |
| G32..00 | Old myocardial infarction |
| G32..11 | Healed myocardial infarction |
| G32..12 | Personal history of myocardial infarction |
| G33..00 | Angina pectoris |
| G330.00 | Angina decubitus |
| G330000 | Nocturnal angina |
| G330z00 | Angina decubitus NOS |
| medcode | description |
| G33z.00 | Angina pectoris NOS |
| G33z000 | Status anginosus |
| G33z100 | Stenocardia |
| G33z200 | Syncope anginosa |
| G33z300 | Angina on effort |
| G33z400 | Ischaemic chest pain |
| G33z500 | Post infarct angina |
| G33z600 | New onset angina |
| G33z700 | Stable angina |
| G33zz00 | Angina pectoris NOS |
| G34..00 | Other chronic ischaemic heart disease |
| G340.00 | Coronary atherosclerosis |
| G340.11 | Triple vessel disease of the heart |
| G340.12 | Coronary artery disease |
| G340000 | Single coronary vessel disease |
| G340100 | Double coronary vessel disease |
| medcode | description |
| G342.00 | Atherosclerotic cardiovascular disease |
| G343.00 | Ischaemic cardiomyopathy |
| G344.00 | Silent myocardial ischaemia |
| G34y.00 | Other specified chronic ischaemic heart disease |
| G34y000 | Chronic coronary insufficiency |
| G34y100 | Chronic myocardial ischaemia |
| G34yz00 | Other specified chronic ischaemic heart disease NOS |
| G34z.00 | Other chronic ischaemic heart disease NOS |
| G34z000 | Asymptomatic coronary heart disease |
| G35..00 | Subsequent myocardial infarction |
| G350.00 | Subsequent myocardial infarction of anterior wall |
| G351.00 | Subsequent myocardial infarction of inferior wall |
| G353.00 | Subsequent myocardial infarction of other sites |
| G35X.00 | Subsequent myocardial infarction of unspecified site |
| G38..00 | Postoperative myocardial infarction |
| G380.00 | Postoperative transmural myocardial infarction anterior wall |
| G381.00 | Postoperative transmural myocardial infarction inferior wall |
| G382.00 | Postoperative transmural myocardial infarction other sites |
| G383.00 | Postoperative transmural myocardial infarction unspec site |
| G384.00 | Postoperative subendocardial myocardial infarction |
| G38z.00 | Postoperative myocardial infarction, unspecified |
| G39..00 | Coronary microvascular disease |
| G3y..00 | Other specified ischaemic heart disease |
| G3z..00 | Ischaemic heart disease NOS |
| Gyu3.00 | [X]Ischaemic heart diseases |
| Gyu3000 | [X]Other forms of angina pectoris |
| Gyu3200 | [X]Other forms of acute ischaemic heart disease |
| Gyu3300 | [X]Other forms of chronic ischaemic heart disease |
| Gyu3400 | [X]Acute transmural myocardial infarction of unspecif site |
| Gyu3500 | [X]Subsequent myocardial infarction of other sites |
| Gyu3600 | [X]Subsequent myocardial infarction of unspecified site |
| PAD codes |  |
| G73..00 | Other peripheral vascular disease |
| G73..11 | Peripheral ischaemic vascular disease |
| G73..12 | Ischaemia of legs |
| G73..13 | Peripheral ischaemia |
| G730.00 | Raynaud's syndrome |
| G730000 | Raynaud's disease |
| G730100 | Raynaud's phenomenon |
| G730111 | Vibratory white finger |
| G730z00 | Raynaud's syndrome NOS |
| G731.00 | Thromboangiitis obliterans |
| G731000 | Buerger's disease |
| G731100 | Presenile gangrene |
| G731z00 | Thromboangiitis obliterans NOS |
| G732.00 | Peripheral gangrene |
| G732000 | Gangrene of toe |
| G732100 | Gangrene of foot |
| G732200 | Gangrene of finger |
| G732300 | Gangrene of thumb |
| G732400 | Gangrene of hand |
| G733.00 | Ischaemic foot |
| G734.00 | Peripheral arterial disease |
| G735.00 | HAVS - Hand-arm vibration syndrome |
| G735.11 | Vibration white finger |
| G73y.00 | Other specified peripheral vascular disease |
| G73y000 | Diabetic peripheral angiopathy |
| G73y100 | Peripheral angiopathic disease EC NOS |
| G73y200 | Acrocyanosis |
| G73y400 | Acroparaesthesia - Schultze's type |
| G73y411 | Schultze's simple acroparaesthesia |
| G73y500 | Acroparaesthesia - Nothnagel's type |
| G73y511 | Nothnagel's vasomotor acroparaesthesia |
| G73y600 | Acroparaesthesia - unspecified |
| G73y700 | Erythrocyanosis |
| G73y800 | Erythromelalgia |
| G73y811 | Erythralgia |
| G73yz00 | Other specified peripheral vascular disease NOS |
| G73z.00 | Peripheral vascular disease NOS |
| G73z000 | Intermittent claudication |
| G73z011 | Claudication |
| G73z012 | Vascular claudication |
| G73zz00 | Peripheral vascular disease NOS |
| Gyu7400 | [X]Other specified peripheral vascular diseases |
| G734.00 | Peripheral arterial disease |
| G73y.00 | Other specified peripheral vascular disease |
| Stroke/TIA codes |  |
| G61..00 | Intracerebral haemorrhage |
| G61..11 | CVA - cerebrovascular accid due to intracerebral haemorrhage |
| G61..12 | Stroke due to intracerebral haemorrhage |
| G610.00 | Cortical haemorrhage |
| G611.00 | Internal capsule haemorrhage |
| G612.00 | Basal nucleus haemorrhage |
| G613.00 | Cerebellar haemorrhage |
| G614.00 | Pontine haemorrhage |
| G615.00 | Bulbar haemorrhage |
| G616.00 | External capsule haemorrhage |
| G618.00 | Intracerebral haemorrhage, multiple localized |
| G619.00 | Lobar cerebral haemorrhage |
| G61X.00 | Intracerebral haemorrhage in hemisphere, unspecified |
| G61X000 | Left sided intracerebral haemorrhage, unspecified |
| G61X100 | Right sided intracerebral haemorrhage, unspecified |
| G61z.00 | Intracerebral haemorrhage NOS |
| G63y.00 | Other precerebral artery occlusion |
| G63y000 | Cerebral infarct due to thrombosis of precerebral arteries |
| G63y100 | Cerebral infarction due to embolism of precerebral arteries |
| G64..00 | Cerebral arterial occlusion |
| G64..11 | CVA - cerebral artery occlusion |
| G64..12 | Infarction - cerebral |
| G64..13 | Stroke due to cerebral arterial occlusion |
| G640.00 | Cerebral thrombosis |
| G640000 | Cerebral infarction due to thrombosis of cerebral arteries |
| G641.00 | Cerebral embolism |
| G641.11 | Cerebral embolus |
| G641000 | Cerebral infarction due to embolism of cerebral arteries |
| G64z.00 | Cerebral infarction NOS |
| G64z.11 | Brainstem infarction NOS |
| G64z.12 | Cerebellar infarction |
| G64z000 | Brainstem infarction |
| G64z100 | Wallenberg syndrome |
| G64z111 | Lateral medullary syndrome |
| G64z200 | Left sided cerebral infarction |
| G64z300 | Right sided cerebral infarction |
| G64z400 | Infarction of basal ganglia |
| G66..00 | Stroke and cerebrovascular accident unspecified |
| G66..11 | CVA unspecified |
| G66..12 | Stroke unspecified |
| G66..13 | CVA - Cerebrovascular accident unspecified |
| G660.00 | Middle cerebral artery syndrome |
| G661.00 | Anterior cerebral artery syndrome |
| G662.00 | Posterior cerebral artery syndrome |
| G663.00 | Brain stem stroke syndrome |
| G664.00 | Cerebellar stroke syndrome |
| G665.00 | Pure motor lacunar syndrome |
| G666.00 | Pure sensory lacunar syndrome |
| G667.00 | Left sided CVA |
| G668.00 | Right sided CVA |
| G676000 | Cereb infarct due cerebral venous thrombosis, nonpyogenic |
| G6W..00 | Cereb infarct due unsp occlus/stenos precerebr arteries |
| G6X..00 | Cerebrl infarctn due/unspcf occlusn or sten/cerebrl artrs |
| Gy62.00 | Rupture of dialysis arteriovenous shunt |
| Gyu..00 | [X]Additional circulatory system disease classificatn terms |
| Gyu0.00 | [X]Acute rheumatic fever |
| Gyu0000 | [X]Other acute rheumatic heart disease |
| Gyu1.00 | [X] Chronic rheumatic heart disease |
| Gyu1000 | [X]Other mitral valve diseases |
| Gyu1100 | [X]Other rheumatic aortic valve diseases |
| Gyu1200 | [X]Other tricuspid valve diseases |
| Gyu1300 | [X]Other multiple valve diseases |
| Gyu1400 | [X]Other specified rheumatic heart diseases |
| Gyu1500 | [X]Multiple valve disease, unspecified |
| Gyu2.00 | [X]Hypertensive diseases |
| Gyu2000 | [X]Other secondary hypertension |
| Gyu2100 | [X]Hypertension secondary to other renal disorders |
| Gyu3.00 | [X]Ischaemic heart diseases |
| Gyu3000 | [X]Other forms of angina pectoris |
| Gyu3100 | [X]Other current complications following acute myocardial infarct |
| Gyu3200 | [X]Other forms of acute ischaemic heart disease |
| Gyu3300 | [X]Other forms of chronic ischaemic heart disease |
| Gyu3400 | [X]Acute transmural myocardial infarction of unspecified site |
| Gyu3500 | [X]Subsequent myocardial infarction of other sites |
| Gyu3600 | [X]Subsequent myocardial infarction of unspecified site |
| Gyu4.00 | [X]Pulmonary heart disease & diseases of pulmonary circulation |
| Gyu4000 | [X]Other specified pulmonary heart diseases |
| Gyu4100 | [X]Other diseases of pulmonary vessels |
| Gyu5.00 | [X]Other forms of heart disease |
| Gyu5000 | [X]Other forms of acute pericarditis |
| Gyu5100 | [X]Other specified diseases of pericardium |
| Gyu5200 | [X]Pericarditis in bacterial diseases classified elsewhere |
| Gyu5300 | [X]Pericarditis in other infectious+parasitic diseases CE |
| Gyu5400 | [X]Pericarditis in other diseases classified elsewhere |
| Gyu5500 | [X]Other nonrheumatic mitral valve disorders |
| Gyu5600 | [X]Other aortic valve disorders |
| Gyu5700 | [X]Other nonrheumatic tricuspid valve disorders |
| Gyu5800 | [X]Other pulmonary valve disorders |
| Gyu5900 | [X]Mitral valve disorders in diseases classified elsewhere |
| Gyu5A00 | [X]Aortic valve disorders in diseases classified elsewhere |
| Gyu5a00 | [X]Other specified cardiac arrhythmias |
| Gyu5b00 | [X]Other ill-defined heart diseases |
| Gyu5B00 | [X]Tricuspid valve disorders/diseases CE |
| Gyu5c00 | [X]Other heart disorders in bacterial diseases CE |
| Gyu5C00 | [X]Pulmonary valve disorders in diseases CE |
| Gyu5D00 | [X]Multiple valve disorders/diseases CE |
| Gyu5d00 | [X]Oth heart disorders/oth infectious+parasitic diseases CE |
| Gyu5e00 | [X]Other heart disorders in other diseases CE |
| Gyu5E00 | [X]Endocarditis,valve unspecified,in diseases CE |
| Gyu5f00 | [X]Nonrheumatic tricuspid valve disorder, unspecified |
| Gyu5F00 | [X]Other acute myocarditis |
| Gyu5G00 | [X]Acute myocarditis, unspecified |
| Gyu5g00 | [X]Cardiovascular disease, unspecified |
| Gyu5H00 | [X]Myocarditis in bacterial diseases classified elsewhere |
| Gyu5J00 | [X]Myocarditis in viral diseases classified elsewhere |
| Gyu5K00 | [X]Myocarditis in other infectious+parasitic diseases CE |
| Gyu5L00 | [X]Myocarditis in other diseases classified elsewhere |
| Gyu5M00 | [X]Other hypertrophic cardiomyopathy |
| Gyu5N00 | [X]Other restrictive cardiomyopathy |
| Gyu5P00 | [X]Other cardiomyopathies |
| Gyu5Q00 | [X]Cardiomyopathy in infectious+parasitic diseases CE |
| Gyu5R00 | [X]Cardiomyopathy in metabolic diseases CE |
| Gyu5S00 | [X]Cardiomyopathy in nutritional diseases CE |
| Gyu5T00 | [X]Cardiomyopathy in other diseases classified elsewhere |
| Gyu5U00 | [X]Other and unspecified atrioventricular block |
| Gyu5V00 | [X]Other and unspecified fascicular block |
| Gyu5W00 | [X]Other and unspecified right bundle-branch block |
| Gyu5X00 | [X]Other specified heart block |
| Gyu5Y00 | [X]Other specified conduction disorders |
| Gyu5Z00 | [X]Other and unspecified premature depolarization |
| Gyu6.00 | [X]Cerebrovascular diseases |
| Gyu6000 | [X]Subarachnoid haemorrhage from other intracranial arteries |
| Gyu6100 | [X]Other subarachnoid haemorrhage |
| Gyu6200 | [X]Other intracerebral haemorrhage |
| Gyu6300 | [X]Cerebrl infarctn due/unspcf occlusn or sten/cerebrl artrs |
| Gyu6400 | [X]Other cerebral infarction |
| Gyu6500 | [X]Occlusion and stenosis of other precerebral arteries |
| Gyu6600 | [X]Occlusion and stenosis of other cerebral arteries |
| Gyu6F00 | [X]Intracerebral haemorrhage in hemisphere, unspecified |
| Gyu6G00 | [X]Cereb infarct due unsp occlus/stenos precerebr arteries |
| G65..00 | Transient cerebral ischaemia |
| G65..11 | Drop attack |
| G65..12 | Transient ischaemic attack |
| G65..13 | Vertebro-basilar insufficiency |
| G650.00 | Basilar artery syndrome |
| G650.11 | Insufficiency - basilar artery |
| G651.00 | Vertebral artery syndrome |
| G651000 | Vertebro-basilar artery syndrome |
| G652.00 | Subclavian steal syndrome |
| G653.00 | Carotid artery syndrome hemispheric |
| G654.00 | Multiple and bilateral precerebral artery syndromes |
| G656.00 | Vertebrobasilar insufficiency |
| G657.00 | Carotid territory transient ischaemic attack |
| G65y.00 | Other transient cerebral ischaemia |
| G65z.00 | Transient cerebral ischaemia NOS |
| G65z000 | Impending cerebral ischaemia |
| G65z100 | Intermittent cerebral ischaemia |
| G65zz00 | Transient cerebral ischaemia NOS |
| ZV12D00 | [V]Personal history of transient ischaemic attack |
| Fyu5500 | [X]Other transnt cerebral ischaemic attacks+related syndroms |

Abbreviations: CHD, coronary heart disease; NOS, not otherwise specified; [X], Cross referenced to specific ICD-10 codes; PAD, peripheral arterial disease; TIA, transient ischaemic attack; CVA, cardiovascular accident

**Read codes used to identify cases of heart failure**

| **Read code** | **Description** |
| --- | --- |
| G58..00 | Heart failure |
| G58..11 | Cardiac failure |
| G580.00 | Congestive heart failure |
| G580.11 | Congestive cardiac failure |
| G580.12 | Right heart failure |
| G580.13 | Right ventricular failure |
| G580.14 | Biventricular failure |
| G580000 | Acute congestive heart failure |
| G580100 | Chronic congestive heart failure |
| G580200 | Decompensated cardiac failure |
| G580300 | Compensated cardiac failure |
| G580400 | Congestive heart failure due to valvular disease |
| G581.00 | Left ventricular failure |
| G581.11 | Asthma - cardiac |
| G581.12 | Pulmonary oedema - acute |
| G581.13 | Impaired left ventricular function |
| G581000 | Acute left ventricular failure |
| G582.00 | Acute heart failure |
| G583.00 | Heart failure with normal ejection fraction |
| G583.11 | HFNEF - heart failure with normal ejection fraction |
| G583.12 | Heart failure with preserved ejection fraction |
| G584.00 | Right ventricular failure |
| G58z.00 | Heart failure NOS |
| G58z.11 | Weak heart |
| G58z.12 | Cardiac failure NOS |
| G1yz100 | Rheumatic left ventricular failure |
| 662f.00 | New York Heart Association classification - class I |
| 662F.00 | Hypertension treatment. started |
| 662G.00 | Hypertensive treatment changed |
| 662g.00 | New York Heart Association classification - class II |
| 662H.00 | Hypertension treatment stopped |
| 662h.00 | New York Heart Association classification - class III |
| 662i.00 | New York Heart Association classification - class IV |
| 585f.00 | Echocardiogram shows left ventricular systolic dysfunction |
| G5yy900 | Left ventricular systolic dysfunction |
| G5yyD00 | Left ventricular cardiac dysfunction |

Abbreviations: NOS, not otherwise specified

**Drug codes used to identify prescription of non-steroidal anti-inflammatory drug**

| **Drug code** | **Generic name** |
| --- | --- |
| 52437979 | Naproxen 250mg tablets |
| 52438979 | Mefenamic acid 500mg tablets |
| 53244979 | Ibuprofen lysine 400mg oral powder sachets |
| 53245979 | Ibuprofen lysine 400mg oral powder sachets |
| 54501979 | Ibuprofen 200mg capsules |
| 59408979 | Indometacin 25mg capsules |
| 60599979 | Etoricoxib 30mg tablets |
| 68279979 | Naproxen 75mg/5ml oral suspension |
| 68284979 | Naproxen 100mg/5ml oral suspension |
| 70267978 | Ibuprofen 200mg tablets |
| 79388978 | Misoprostol 400microgram tablets |
| 79449979 | Naproxen 500mg/5ml oral suspension |
| 79451979 | Naproxen 250mg/5ml oral suspension |
| 79837979 | Indometacin 25mg/5ml oral solution |
| 81659998 | Ibuprofen 200mg effervescent tablets |
| 81664998 | Ibuprofen 10% gel |
| 81960998 | Ketoprofen 200mg modified-release capsules |
| 82031998 | Celecoxib 400mg capsules |
| 82272998 | Ibuprofen 5% spray |
| 82307998 | Tenoxicam 20mg tablets |
| 82366998 | Ibuprofen lysine 200mg tablets |
| 82686998 | Ibuprofen 5% gel |
| 82872978 | Meloxicam 7.5mg orodispersible tablets sugar free |
| 82874978 | Meloxicam 15mg orodispersible tablets sugar free |
| 82923998 | Ketoprofen 150mg modified-release capsules |
| 82924998 | Ketoprofen 200mg modified-release capsules |
| 82925998 | Ketoprofen 100mg modified-release capsules |
| 82926998 | Ketoprofen 100mg suppositories |
| 82927998 | Ketoprofen 100mg capsules |
| 82928998 | Ketoprofen 50mg capsules |
| 83070998 | Naproxen 200mg/5ml oral suspension |
| 83154998 | Ibuprofen 400mg capsules |
| 83155998 | Ibuprofen 200mg capsules |
| 83433998 | Ibuprofen 400mg tablets |
| 83434998 | Aspirin 500mg effervescent tablets sugar free |
| 83445998 | Piroxicam 20mg orodispersible tablets sugar free |
| 83471998 | Ibuprofen 800mg modified-release tablets |
| 83494998 | Ibuprofen sodium dihydrate 200mg tablets |
| 83497998 | Ibuprofen sodium dihydrate 200mg tablets |
| 83500998 | Ibuprofen 200mg tablets |
| 83608998 | Etodolac 600mg modified-release tablets |
| 83611998 | Flurbiprofen 200mg modified release capsules |
| 83984998 | Naproxen 250mg gastro-resistant tablets |
| 84019998 | Ibuprofen 5% gel |
| 84058998 | Ibuprofen 5% cream |
| 84104998 | Ibuprofen 200mg/5ml oral suspension |
| 84128998 | Etoricoxib 30mg tablets |
| 84129998 | Etoricoxib 30mg tablets |
| 84153998 | Ibuprofen 400mg tablets |
| 84155998 | Ibuprofen 200mg tablets |
| 84160998 | Ibuprofen 200mg tablets |
| 84174998 | Ibuprofen 5% gel |
| 84433998 | Ibuprofen 200mg capsules |
| 84434998 | Ibuprofen lysine 400mg tablets |
| 84435998 | Ibuprofen 400mg capsules |
| 84437998 | Ibuprofen lysine 200mg tablets |
| 84490998 | Ibuprofen 200mg tablets |
| 84553998 | Ibuprofen 200mg capsules |
| 84554998 | Ibuprofen 200mg tablets |
| 84555998 | Ibuprofen 200mg tablets |
| 84973998 | Indometacin oral solution |
| 85154998 | Diclofenac sodium 25mg gastro-resistant tablets |
| 85272998 | Naproxen oral solution |
| 85428998 | Etodolac 600mg modified-release tablets |
| 85741998 | Ibuprofen 400mg capsules |
| 85783998 | Ibuprofen 400mg capsules |
| 85891979 | Ibuprofen lysine 200mg tablets |
| 85953998 | Phenylbutazone 100mg tablets |
| 86170998 | Ibuprofen lysine 400mg tablets |
| 86171998 | Ibuprofen 400mg tablets |
| 86329998 | Ibuprofen 10mg/2ml solution for infusion ampoules |
| 86594998 | Ibuprofen lysine 200mg tablets |
| 86624998 | Dexibuprofen 400mg tablets |
| 86628998 | Dexibuprofen 300mg tablets |
| 86629998 | Dexibuprofen 400mg tablets |
| 86635998 | Dexibuprofen 300mg tablets |
| 86940998 | Ibuprofen 5% gel |
| 86953998 | Ibuprofen 200mg tablets |
| 87070998 | Ibuprofen 300mg modified-release capsules |
| 87413998 | Ketoprofen 50mg capsules |
| 87936998 | Aspirin 500mg granules sachets sugar free |
| 88047997 | Ketoprofen 200mg modified-release capsules |
| 88047998 | Ketoprofen 100mg modified-release capsules |
| 88138998 | Naproxen 500mg gastro-resistant tablets |
| 88139998 | Naproxen 250mg gastro-resistant tablets |
| 88143998 | Ibuprofen 5% gel |
| 88145996 | Ibuprofen 400mg tablets |
| 88145998 | Ibuprofen 5% gel |
| 88174998 | Ibuprofen 200mg capsules |
| 88204998 | Ibuprofen 5% mousse |
| 88205998 | Ibuprofen 5% foam |
| 88228998 | Ibuprofen lysine 200mg tablets |
| 88233998 | Ibuprofen 200mg tablets |
| 88284998 | Ibuprofen 400mg tablets |
| 88442998 | Mefenamic acid 250mg capsules |
| 88455998 | Indometacin 75mg modified-release capsules |
| 88527997 | Ibuprofen 200mg capsules |
| 88527998 | Ibuprofen 200mg tablets |
| 88817998 | Tolfenamic acid 200mg tablets |
| 88943998 | Ketoprofen 200mg modified release capsules |
| 88970998 | Etoricoxib 60mg tablets |
| 88977998 | Etoricoxib 120mg tablets |
| 89014998 | Ibuprofen 200mg modified-release capsules |
| 89117998 | Ibuprofen 200mg tablets |
| 89137997 | Ketoprofen 200mg modified release capsules |
| 89139998 | Ibuprofen 5% gel |
| 89217998 | Aspirin 300mg gastro-resistant tablets |
| 89398979 | Etoricoxib 90mg tablets |
| 89404979 | Etoricoxib 60mg tablets |
| 89472997 | Ibuprofen 5% gel |
| 89479998 | Tolfenamic acid 200mg capsules |
| 89484997 | Tolfenamic acid 200mg tablets |
| 89484998 | Tolfenamic acid 200mg capsule |
| 89499998 | Ibuprofen 10% gel |
| 89572998 | Etodolac 300mg capsules |
| 89580998 | Ibuprofen 200mg tablets |
| 89593997 | Ibuprofen 400mg tablets |
| 89621998 | Ibuprofen 200mg orodispersible tablets sugar free |
| 89691997 | Ibuprofen 400mg tablets |
| 89691998 | Ibuprofen 200mg tablets |
| 89760998 | Ibuprofen 300mg modified-release capsules |
| 89801998 | Flurbiprofen 8.75mg lozenges |
| 89890998 | Ibuprofen lysine 200mg tablets |
| 89898998 | Codeine 8mg with aspirin 500mg soluble tablets |
| 89909998 | Ketoprofen 200mg modified release capsules |
| 89966998 | Ketoprofen 2.5% gel |
| 89993998 | Ibuprofen 5% gel |
| 90116998 | Ibuprofen 200mg tablets |
| 90125998 | Ibuprofen 200mg tablets |
| 90278998 | Aspirin 500mg effervescent tablets sugar free |
| 90351997 | Meloxicam 15mg tablets |
| 90351998 | Meloxicam 7.5mg tablets |
| 90361997 | Meloxicam 15mg tablets |
| 90361998 | Meloxicam 7.5mg tablets |
| 90368997 | Celecoxib 200mg capsules |
| 90368998 | Celecoxib 100mg capsules |
| 90377997 | Aspirin 500mg effervescent tablets sugar free |
| 90377998 | Aspirin 300mg effervescent tablets sugar free |
| 90635998 | Aceclofenac 100mg tablets |
| 90636998 | Aceclofenac 100mg tablets |
| 90709998 | Flurbiprofen 8.75mg lozenges |
| 90846998 | Ketoprofen 200mg modified-release capsules |
| 90869998 | Ibuprofen 400mg tablets |
| 90954998 | Flurbiprofen 8.75mg lozenges |
| 91081996 | Ibuprofen 200mg tablets |
| 91081997 | Ibuprofen 200mg capsules |
| 91081998 | Ibuprofen 400mg granules |
| 91105998 | Indometacin 25mg modified-release capsules |
| 91109997 | Mefenamic acid 500mg tablets |
| 91109998 | Mefenamic acid 250mg capsules |
| 91120998 | Ibuprofen 200mg tablets |
| 91155998 | Ibuprofen 10% gel |
| 91315998 | Ketoprofen 2.5% gel |
| 91421998 | Dexketoprofen 25mg tablets |
| 91438979 | Ibuprofen 10% gel |
| 91442979 | Piroxicam 0.5% gel |
| 91443979 | Piroxicam 0.5% gel |
| 91446979 | Piroxicam 0.5% gel |
| 91447979 | Piroxicam 0.5% gel |
| 91451979 | Piroxicam 0.5% gel |
| 91463997 | Piroxicam 20mg capsules |
| 91463998 | Piroxicam 10mg capsules |
| 91466979 | Ketoprofen 2.5% gel |
| 91475979 | Ibuprofen 5% gel |
| 91479979 | Celecoxib 100mg capsules |
| 91486979 | Celecoxib 200mg capsules |
| 91502998 | Indometacin 75mg modified release capsules |
| 91517998 | Piroxicam 0.5% gel |
| 91523998 | Ibuprofen 10% gel |
| 91581997 | Celecoxib 200mg capsules |
| 91581998 | Celecoxib 100mg capsules |
| 91682979 | Tenoxicam 20mg tablets |
| 91713979 | Piroxicam 20mg orodispersible tablets sugar free |
| 91751979 | Naproxen 250mg tablets |
| 91752979 | Naproxen 250mg tablets |
| 91757979 | Naproxen 250mg tablets |
| 91763979 | Mefenamic acid 500mg tablets |
| 91770979 | Mefenamic acid 250mg capsules |
| 91774979 | Ketoprofen 200mg modified-release capsules |
| 91774998 | Indometacin 75mg modified release capsules |
| 91777998 | Ibuprofen 10% gel |
| 91778979 | Ketoprofen 200mg modified-release capsules |
| 91782979 | Ketoprofen 100mg modified-release capsules |
| 91815997 | Mefenamic acid 500mg tablets |
| 91815998 | Mefenamic acid 250mg capsules |
| 91841998 | Aspirin 300mg orodispersible tablets sugar free |
| 91843979 | Ibuprofen 400mg tablets |
| 91850979 | Ibuprofen 400mg tablets |
| 91851979 | Ibuprofen 400mg tablets |
| 91854979 | Ibuprofen 400mg tablets |
| 91856979 | Ibuprofen 200mg tablets |
| 91863979 | Ibuprofen 200mg tablets |
| 91864979 | Ibuprofen 200mg tablets |
| 91877998 | Ketoprofen 200mg modified release capsules |
| 91920997 | Mefenamic acid 500mg tablets |
| 91920998 | Mefenamic acid 250mg capsules |
| 91965979 | Aspirin 300mg tablets |
| 91988998 | Etoricoxib 90mg tablets |
| 91989998 | Etoricoxib 60mg tablets |
| 91990998 | Etoricoxib 120mg tablets |
| 91991998 | Etoricoxib 90mg tablets |
| 92092990 | Ketoprofen 2.5% gel |
| 92112998 | Ketoprofen 200mg modified-release capsules |
| 92113998 | Indometacin 75mg modified release capsules |
| 92158998 | Tiaprofenic acid 300mg tablets |
| 92169998 | Ibuprofen 200mg capsules |
| 92189998 | Piroxicam betadex 20mg tablets |
| 92290998 | Ibuprofen 200mg orodispersible tablets sugar free |
| 92550990 | Mefenamic acid 500mg tablets |
| 92551990 | Mefenamic acid 250mg capsules |
| 92671998 | Aspirin 500mg modified-release tablets |
| 92706998 | Aspirin 300mg gastro-resistant tablets |
| 92738990 | Naproxen 500mg gastro-resistant tablets |
| 92778998 | Aspirin 300mg orodispersible tablets sugar free |
| 92801998 | Piroxicam 10mg capsules |
| 92851998 | Tenoxicam 20mg injection plus diluent |
| 92863998 | Etodolac 600mg modified-release tablets |
| 92864998 | Etodolac 600mg modified-release tablets |
| 92950996 | Naproxen 500mg gastro-resistant tablets |
| 92950997 | Naproxen 250mg tablets |
| 92950998 | Naproxen 500mg tablets |
| 92953997 | Ketoprofen 100mg capsules |
| 92954997 | Indometacin 75mg modified release capsules |
| 92954998 | Indometacin 25mg capsules |
| 92958998 | Indometacin 1mg injection (powder for reconstitution) |
| 92965998 | Ibuprofen 400mg tablets |
| 93029998 | Naproxen 500mg modified release tablets |
| 93089997 | Ketoprofen 200mg modified-release capsules |
| 93089998 | Ketoprofen 100mg modified-release capsules |
| 93099997 | Aspirin 300mg modified release tablets |
| 93135996 | Naproxen 500mg modified-release tablets |
| 93135997 | Naproxen 375mg modified-release tablet |
| 93135998 | Naproxen 500mg granules |
| 93152998 | Ketoprofen 100mg/2ml injection |
| 93169996 | Naproxen 375mg gastro-resistant tablets |
| 93169997 | Naproxen 500mg gastro-resistant tablets |
| 93169998 | Naproxen 250mg gastro-resistant tablets |
| 93170997 | Naproxen 500mg gastro-resistant tablets |
| 93170998 | Naproxen 250mg gastro-resistant tablets |
| 93218998 | Ibuprofen 200mg tablets |
| 93235990 | Ibuprofen 10% gel |
| 93247998 | Acemetacin 60mg capsules |
| 93261998 | Nabumetone 500mg dispersible tablets sugar free |
| 93267998 | Naproxen 375mg tablets |
| 93272996 | Ibuprofen lysine 400mg tablets |
| 93272997 | Ibuprofen 200mg tablets |
| 93272998 | Ibuprofen 200mg tablets |
| 93351990 | Indometacin 25mg capsules |
| 93368998 | Aspirin 300mg dispersible tablets |
| 93579990 | Meloxicam 15mg tablets |
| 93580990 | Meloxicam 7.5mg tablets |
| 93625998 | Ibuprofen 5% cream |
| 93626996 | Ibuprofen 5% spray |
| 93626997 | Ibuprofen 5% gel |
| 93626998 | Ibuprofen 5% cream |
| 93688992 | Aspirin s/r 500 mg tab |
| 93698990 | Meloxicam 7.5mg tablets |
| 93726990 | Meloxicam 7.5mg tablets |
| 93731992 | Aspirin disp 500 mg tab |
| 93756990 | Ibuprofen 400mg tablets |
| 93866998 | Ibuprofen 200mg tablets |
| 94020992 | Aspirin soluble 600 mg tab |
| 94073992 | Aspirin 600 mg sup |
| 94152998 | Ibuprofen 10% gel |
| 94165992 | Fenoprofen 300mg tablets |
| 94213998 | Aspirin 600mg tablets |
| 94214997 | Aspirin 300mg gastro-resistant tablets |
| 94214998 | Aspirin 324mg gastro-resistant tablets |
| 94215996 | Aspirin 300mg orodispersible tablets sugar free |
| 94215997 | Aspirin 300mg orodispersible tablets sugar free |
| 94216997 | Aspirin 300mg modified-release tablets |
| 94216998 | Aspirin 324mg modified-release tablets |
| 94240992 | Ibuprofen 200mg tablets |
| 94254998 | Aspirin 300mg gastro-resistant tablets |
| 94257998 | Piroxicam 0.5% gel |
| 94258997 | Piroxicam 0.5% gel |
| 94258998 | Piroxicam 0.5% gel |
| 94262998 | Aspirin 500mg with papaveretum 7.71mg dispersible tablets |
| 94352998 | Ketoprofen 100mg/2ml solution for injection ampoules |
| 94437990 | Piroxicam 0.5% gel |
| 94459990 | Ibuprofen 400mg tablets |
| 94489996 | Ketoprofen 150mg modified-release capsules |
| 94489997 | Ketoprofen 200mg modified-release capsules |
| 94489998 | Ketoprofen 100mg modified-release capsules |
| 94513997 | Aspirin 300mg effervescent tablets |
| 94514996 | Tenoxicam 20mg effervescent tablets |
| 94514997 | Tenoxicam 20mg/sachet granules |
| 94514998 | Tenoxicam 20mg tablets |
| 94515996 | Tenoxicam 20mg effervescent tablets |
| 94515998 | Tenoxicam 20mg tablets |
| 94589997 | Aspirin 300mg effervescent tablets |
| 94589998 | Aspirin 100mg effervescent tablets |
| 94607996 | Ketorolac 10mg/1ml solution for injection ampoules |
| 94607997 | Ketorolac 10mg tablets |
| 94607998 | Ketorolac 30mg/1ml solution for injection ampoules |
| 94608996 | Ketorolac trometamol 10mg/1ml injection |
| 94608997 | Ketorolac trometamol 10mg tablets |
| 94608998 | Ketorolac 30mg/1ml solution for injection ampoules |
| 94626990 | Ibuprofen 400mg tablets |
| 94631998 | Misoprostol 200microgram tablets |
| 94632998 | Misoprostol 200microgram vaginal tablets |
| 94651992 | Ibuprofen 400mg tablets |
| 94667992 | Aspirin m/f 324 mg tab |
| 94668992 | Aspirin 325 mg cap |
| 94671992 | Aspirin 500 mg sup |
| 94674992 | Aspirin disp 600 mg tab |
| 94678990 | Ibuprofen 400mg tablets |
| 94678992 | Aspirin soluble 400 mg tab |
| 94679992 | Aspirin soluble 500 mg tab |
| 94709997 | Aspirin 300mg effervescent tablets sugar free |
| 94709998 | Aspirin 500mg effervescent tablets sugar free |
| 94743992 | Brufen sup |
| 94759998 | Aspirin 500mg with cyclizine 25mg effervescent tablets |
| 94784998 | Flurbiprofen 200mg modified-release capsules |
| 94798998 | Flurbiprofen 200mg modified release capsules |
| 94805997 | Ibuprofen 600mg effervescent granules sachets |
| 94805998 | Ibuprofen 800mg modified-release tablets |
| 94809997 | Piroxicam 20mg dispersible tablets |
| 94809998 | Piroxicam 10mg dispersible tablets |
| 94832996 | Indometacin 25mg modified release tablets |
| 94832997 | Indometacin 50mg modified release tablets |
| 94832998 | Indometacin 75mg modified release tablets |
| 94874998 | Ibuprofen 800mg tablets |
| 94875998 | Ibuprofen 800mg tablets |
| 94887998 | Ibuprofen 200mg orodispersible tablets sugar free |
| 94907997 | Nabumetone 500mg/5ml oral suspension sugar free |
| 94907998 | Nabumetone 500mg tablets |
| 94914997 | Nabumetone 500mg/5ml suspension |
| 94914998 | Nabumetone 500mg tablets |
| 94916998 | Ibuprofen 5% foam |
| 94928998 | Tiaprofenic acid 300mg modified release capsules |
| 95006998 | Ketoprofen 2.5% gel |
| 95013998 | Ketoprofen 2.5% gel |
| 95014992 | Fenoprofen 300mg tablets |
| 95061998 | Dexketoprofen 25mg tablets |
| 95075998 | Indometacin 25mg modified-release tablets |
| 95093996 | Naproxen 500mg gastro-resistant tablets |
| 95093997 | Naproxen 375mg gastro-resistant tablets |
| 95093998 | Naproxen 250mg gastro-resistant tablets |
| 95143992 | Ibuprofen 200mg capsules |
| 95167996 | Tiaprofenic acid 300mg sachets |
| 95167997 | Tiaprofenic acid 300mg tablets |
| 95167998 | Tiaprofenic acid 200mg tablets |
| 95172990 | Ibuprofen 10% gel |
| 95191990 | Piroxicam 0.5% gel |
| 95212992 | Aspirin 500mg modified release tablets |
| 95227997 | Sulindac 200mg tablets |
| 95227998 | Sulindac 100mg tablets |
| 95340990 | Ibuprofen 200mg tablets |
| 95347990 | Ibuprofen 400mg tablets |
| 95348990 | Ibuprofen 200mg tablets |
| 95351990 | Aspirin 300mg dispersible tablets |
| 95496990 | Piroxicam 0.5% gel |
| 95496998 | Piroxicam 10mg capsules |
| 95497998 | Piroxicam 20mg suppositories |
| 95498996 | Piroxicam 20mg orodispersible tablets sugar free |
| 95498997 | Piroxicam 20mg capsules |
| 95498998 | Piroxicam 10mg capsules |
| 95539997 | Phenylbutazone 200mg tablets |
| 95539998 | Phenylbutazone 100mg tablets |
| 95540998 | Phenylbutazone 100mg gastro-resistant tablets |
| 95541997 | Phenylbutazone 200mg tablets |
| 95541998 | Phenylbutazone 100mg tablets |
| 95611990 | Ketoprofen 2.5% gel |
| 95753998 | Naproxen sodium 275mg tablets |
| 95754997 | Naproxen 125mg/5ml oral suspension |
| 95754998 | Naproxen 500mg suppositories |
| 95909996 | Mefenamic acid 50mg/5ml oral suspension |
| 95909997 | Mefenamic acid 500mg tablets |
| 95909998 | Mefenamic acid 250mg dispersible tablet |
| 95911992 | Aspirin 300mg dispersible tablets |
| 95992990 | Ibuprofen 5% gel |
| 96035996 | Ketoprofen 100mg suppositories |
| 96035997 | Ketoprofen 100mg capsules |
| 96035998 | Ketoprofen 50mg capsules |
| 96310989 | Ibuprofen 400mg tablets |
| 96310990 | Ibuprofen 200mg tablets |
| 96369990 | Ibuprofen 200mg tablets |
| 96405996 | Ibuprofen 600mg tablets |
| 96405997 | Ibuprofen 400mg tablets |
| 96405998 | Ibuprofen 200mg tablets |
| 96407989 | Indometacin 50mg capsules |
| 96407990 | Indometacin 25mg capsules |
| 96414990 | Aspirin 300mg dispersible tablets |
| 96418989 | Ibuprofen 400mg tablets |
| 96451997 | Naproxen 500mg tablets |
| 96451998 | Naproxen 250mg tablets |
| 96452997 | Naproxen 500mg tablets |
| 96452998 | Naproxen 250mg tablets |
| 96495996 | Flurbiprofen 100mg suppositories |
| 96495997 | Flurbiprofen 100mg tablets |
| 96495998 | Flurbiprofen 50mg tablets |
| 96555996 | Fenoprofen 600mg tablets |
| 96555997 | Fenoprofen 300mg tablets |
| 96555998 | Fenoprofen 200mg tablet |
| 96566989 | Aspirin 300mg dispersible tablets |
| 96566990 | Aspirin 300mg tablets |
| 96569992 | Aspirin sr 300 mg tab |
| 96583990 | Ibuprofen 5% gel |
| 96625988 | Naproxen 500mg gastro-resistant tablets |
| 96841990 | Piroxicam 20mg dispersible tablets |
| 96842989 | Piroxicam 20mg capsules |
| 96851990 | Mefenamic acid 250mg capsules |
| 96879990 | Naproxen 250mg gastro-resistant tablets |
| 96921998 | Nabumetone 500mg dispersible tablets |
| 96939989 | Ibuprofen 400mg tablets |
| 96939990 | Ibuprofen 200mg tablets |
| 96961989 | Mefenamic acid 500mg tablets |
| 96961990 | Mefenamic acid 250mg capsules |
| 97056996 | Ibuprofen 600mg tablets |
| 97056997 | Ibuprofen 400mg tablets |
| 97056998 | Ibuprofen 200mg tablets |
| 97088992 | Aspirin 324mg gastro-resistant tablets |
| 97100989 | Naproxen 500mg gastro-resistant tablets |
| 97100990 | Naproxen 250mg gastro-resistant tablets |
| 97104988 | Ibuprofen 600mg tablets |
| 97104989 | Ibuprofen 400mg tablets |
| 97104990 | Ibuprofen 200mg tablets |
| 97106997 | Ibuprofen 400mg tablets |
| 97107997 | Ibuprofen 400mg tablets |
| 97111990 | Mefenamic acid 250mg capsules |
| 97114990 | Indometacin 75mg modified-release capsules |
| 97180989 | Naproxen 500mg gastro-resistant tablets |
| 97180990 | Naproxen 250mg gastro-resistant tablets |
| 97181990 | Aspirin 300mg gastro-resistant tablets |
| 97305992 | Dihydrocodeine tartrate/aspirin 300 mg tab |
| 97356998 | Indometacin 75mg modified-release capsules |
| 97357996 | Indometacin 100mg suppositories |
| 97358997 | Ibuprofen 400mg tablets |
| 97358998 | Ibuprofen 200mg tablets |
| 97537989 | Aspirin 300mg gastro-resistant tablets |
| 97550988 | Naproxen 250mg gastro-resistant tablets |
| 97551989 | Ibuprofen 400mg tablets |
| 97551990 | Ibuprofen 200mg tablets |
| 97564998 | Naproxen sodium 275mg tablets |
| 97565998 | Naproxen 500mg tablets |
| 97566996 | Naproxen 500mg suppositories |
| 97566997 | Naproxen 125mg/5ml suspension |
| 97566998 | Naproxen 250mg tablets |
| 97593996 | Ibuprofen 600mg tablets |
| 97594997 | Ibuprofen 400mg tablets |
| 97594998 | Ibuprofen 200mg tablets |
| 97641992 | Ketorolac 30mg/1ml solution for injection ampoules |
| 97657997 | Naproxen 500mg tablets |
| 97657998 | Naproxen 250mg tablets |
| 97658997 | Naproxen 500mg modified release tablets |
| 97658998 | Naproxen 375mg modified release tablets |
| 97668998 | Piroxicam 20mg/1ml injection |
| 97674989 | Ibuprofen 400mg tablets |
| 97674990 | Ibuprofen 200mg tablets |
| 97678997 | Ibuprofen 400mg tablets |
| 97700990 | Naproxen 500mg gastro-resistant tablets |
| 97712997 | Naproxen 500mg tablets |
| 97712998 | Naproxen 250mg tablets |
| 97746990 | Ketoprofen 200mg modified-release capsules |
| 97748998 | Piroxicam 20mg/1ml solution for injection ampoules |
| 97902990 | Piroxicam 20mg dispersible tablets |
| 97906996 | Ibuprofen 600mg tablets |
| 97906997 | Ibuprofen 400mg tablets |
| 97906998 | Ibuprofen 200mg tablets |
| 97918989 | Aspirin 300mg dispersible tablets |
| 98040988 | Ketoprofen 100mg capsules |
| 98041990 | Indometacin 50mg capsules |
| 98127989 | Mefenamic acid 500mg tablets |
| 98127990 | Mefenamic acid 250mg capsules |
| 98134989 | Flurbiprofen 50mg tablets |
| 98137998 | Ibuprofen 10% gel |
| 98142989 | Aspirin 300mg dispersible tablets |
| 98150990 | Ibuprofen 600mg tablets |
| 98151990 | Ibuprofen 600mg tablets |
| 98166996 | Piroxicam 20mg orodispersible tablets sugar free |
| 98166997 | Piroxicam 20mg dispersible tablets |
| 98166998 | Piroxicam 10mg dispersible tablets |
| 98280998 | Aspirin & metoclopramide 450mg+5mg effervescent tablets |
| 98399998 | Indometacin 75mg modified-release capsules |
| 98419997 | Aspirin 300mg dispersible tablets |
| 98419998 | Aspirin 300mg tablets |
| 98426989 | Piroxicam 20mg capsules |
| 98426990 | Piroxicam 10mg capsules |
| 98429998 | Ibuprofen & codeine phosphate 300mg+20mg modified release tab |
| 98495989 | Mefenamic acid 500mg tablets |
| 98495990 | Mefenamic acid 250mg capsules |
| 98513990 | Aspirin 300mg tablets |
| 98515998 | Ibuprofen 600mg tablets |
| 98516998 | Ibuprofen 400mg tablets |
| 98528990 | Ibuprofen 600mg tablets |
| 98529988 | Ibuprofen 600mg tablets |
| 98529989 | Ibuprofen 400mg tablets |
| 98529990 | Ibuprofen 200mg tablets |
| 98530988 | Ibuprofen 600mg tablets |
| 98530989 | Ibuprofen 400mg tablets |
| 98530990 | Ibuprofen 200mg tablets |
| 98555989 | Ibuprofen 400mg tablets |
| 98555990 | Ibuprofen 200mg tablets |
| 98578998 | Ibuprofen 300mg modified-release capsules |
| 98592988 | Aspirin 300mg dispersible tablets |
| 98600989 | Ketoprofen 200mg modified-release capsules |
| 98621989 | Ketoprofen 200mg modified-release capsules |
| 98654998 | Mefenamic acid 50mg/5ml paediatric suspension |
| 98671988 | Indometacin 50mg capsules |
| 98671989 | Indometacin 25mg capsules |
| 98671990 | Indometacin 75mg modified-release capsules |
| 98672989 | Indometacin 25mg capsules |
| 98672990 | Indometacin 50mg capsules |
| 98673988 | Ibuprofen 200mg tablets |
| 98673989 | Ibuprofen 400mg tablets |
| 98673990 | Ibuprofen 600mg tablets |
| 98674988 | Naproxen 250mg gastro-resistant tablets |
| 98674989 | Naproxen 500mg gastro-resistant tablets |
| 98674990 | Naproxen 250mg tablets |
| 98693998 | Acemetacin 60mg capsules |
| 98758998 | Ketoprofen 200mg modified release capsules |
| 98764998 | Ibuprofen 200mg modified-release capsules |
| 98779998 | Ketoprofen 100mg suppositories |
| 98907998 | Naproxen 500mg/sachet granules |
| 99334997 | Aspirin 600mg gastro-resistant tablets |
| 99334998 | Aspirin 300mg gastro-resistant tablets |
| 99442989 | Piroxicam 20mg capsules |
| 99442990 | Piroxicam 10mg capsules |
| 99444989 | Piroxicam 20mg capsules |
| 99444990 | Piroxicam 10mg capsules |
| 99445989 | Piroxicam 20mg capsules |
| 99445990 | Piroxicam 10mg capsules |
| 99466996 | Ibuprofen 400mg tablets |
| 99466997 | Ibuprofen 200mg tablets |
| 99482998 | Piroxicam betadex 20mg tablets |
| 99516998 | Tiaprofenic acid 300mg modified-release capsules |
| 99517989 | Mefenamic acid 500mg tablets |
| 99517990 | Mefenamic acid 250mg capsules |
| 99519989 | Mefenamic acid 500mg tablets |
| 99519990 | Mefenamic acid 250mg capsules |
| 99520989 | Mefenamic acid 500mg tablets |
| 99520990 | Mefenamic acid 250mg capsules |
| 99535996 | Indometacin 25mg/5ml sugar free suspension |
| 99535997 | Indometacin 50mg capsules |
| 99535998 | Indometacin 25mg capsules |
| 99539996 | Indometacin 100mg suppositories |
| 99539997 | Indometacin 50mg capsules |
| 99539998 | Indometacin 25mg capsules |
| 99550989 | Indometacin 50mg capsules |
| 99550990 | Indometacin 25mg capsules |
| 99551990 | Indometacin 25mg capsules |
| 99552988 | Indometacin 100mg suppositories |
| 99552989 | Indometacin 50mg capsules |
| 99552990 | Indometacin 25mg capsules |
| 99553989 | Indometacin 75mg modified-release capsules |
| 99553990 | Indometacin 25mg capsules |
| 99557988 | Ibuprofen 600mg tablets |
| 99557989 | Ibuprofen 400mg tablets |
| 99557990 | Ibuprofen 200mg tablets |
| 99558988 | Ibuprofen 400mg tablets |
| 99558989 | Ibuprofen 600mg tablets |
| 99558990 | Ibuprofen 200mg tablets |
| 99621996 | Flurbiprofen 100mg suppositories |
| 99621997 | Flurbiprofen 100mg tablets |
| 99621998 | Flurbiprofen 50mg tablets |
| 99652997 | Fenoprofen 600mg tablets |
| 99652998 | Fenoprofen 300mg tablets |
| 99653996 | Piroxicam 20mg suppositories |
| 99653997 | Piroxicam 20mg capsules |
| 99653998 | Piroxicam 10mg capsules |
| 99728988 | Naproxen 500mg gastro-resistant tablets |
| 99728989 | Naproxen 500mg gastro-resistant tablets |
| 99728990 | Naproxen 250mg tablets |
| 99730989 | Naproxen 500mg gastro-resistant tablets |
| 99730990 | Naproxen 250mg tablets |
| 99731989 | Naproxen 500mg gastro-resistant tablets |
| 99731990 | Naproxen 250mg tablets |
| 99807988 | Aspirin 300mg tablets |
| 99807989 | Aspirin 300mg dispersible tablets |
| 99808988 | Aspirin 300mg tablets |
| 99808989 | Aspirin 300mg dispersible tablets |
| 99810989 | Aspirin 300mg dispersible tablets |
| 99823997 | Sulindac 200mg tablets |
| 99823998 | Sulindac 100mg tablets |
| 99824998 | Aspirin 300mg tablets |
| 99868998 | Ibuprofen 200mg tablets |
| 99876992 | Aspirin 325 mg tab |

**Read code used to detect glycaemic abnormality**

**Note**: Fructosamine is an alternative test of assessing glucose abnormality in patients where HbA1c cannot be reliably measured

| **Read code** | **Description** |
| --- | --- |
| **Sickle cell** | |
| D1060.00 | Sickle cell anaemia of unspecific type |
| D1061.00 | Sickle cell anaemia with no crisis |
| D1062.00 | Sickle cell anaemia with crisis |
| D1063.00 | Sickle cell anaemia with haemoglobin C disease |
| D1064.00 | Sickle cell anaemia with haemoglobin D disease |
| D1065.00 | Sickle cell anaemia with haemoglobin E disease |
| D106z.00 | Sickle cell anaemia NOS |
| **Test result code** | |
| 42D4.00 | Sickle cell present |
| **Thalassaemia** | |
| D1040.00 | Thalassaemia major NEC |
| D1041.00 | Thalassaemia minor NEC |
| D1042.00 | Thalassaemia with haemoglobin S disease |
| D1043.00 | Alpha Thalassaemia |
| D1046.00 | Beta intermediate Thalassaemia |
| D1047.00 | Beta major Thalassaemia |
| D1048.00 | Beta minor Thalassaemia |
| D104z.00 | Thalassaemia NOS |
| **Lab test code** | |
| 44TD.00 | Fructosamine |

Abbreviations: NEC, not elsewhere classified; NOS, not otherwise specified
